# MAGL-18c attenuates LPS-induced sepsis-associated liver injury by inhibiting TGF-β/Smad signaling and remodeling medium- and long-chain fatty acid metabolism

**DOI:** 10.3389/fimmu.2026.1712807

**Published:** 2026-02-05

**Authors:** Ying Wang, Meijia Li, Zixia Liang, Yunyu Wang, Honghua Li, Shirong Li, Xu Jiao, Na Guo, Guoxin Dai, Guimin Zhang, Xiaoyan Lu, Jingchun Yao

**Affiliations:** 1 School of Medicine and Pharmacy, Ocean University of China, Qingdao, China; 2 School of Chinese Materia Medica, Tianjin University of Traditional Chinese Medicine, Tianjin, China; 3 School of Traditional Chinese Medicine, Guangdong Pharmaceutical University, Guangzhou, China; 4 School of Pharmacy, Medical College, Qingdao University, Qingdao, Guangdong, China; 5 State Key Laboratory of Integration and Innovation of Classic Formula and Modern Chinese Medicine, Lunan Pharmaceutical Group Co. Ltd., Linyi, China; 6 Lunan Better Pharmaceutical Co., Ltd., Linyi, China

**Keywords:** LCFAs, MAGL-18c, MCFAs, mitochondrial dysfunction, SALI, TGF-β

## Abstract

**Introduction:**

Sepsis is a major global health burden associated with high mortality and multiple organ dysfunction, among which liver injury is a key determinant of poor prognosis. However, effective therapeutic strategies for sepsis-associated liver injury (SALI) remain limited.

**Methods:**

In this study, we investigated the protective effects of MAGL-18c, a novel monoacylglycerol lipase (MAGL) inhibitor, on lipopolysaccharide (LPS)-induced SALI. Hepatic inflammation, apoptosis, mitochondrial function, and lipid metabolism were assessed using liquid chromatography–mass spectrometry (LC-MS), Western blotting, real-time quantitative PCR (qPCR), immunohistochemistry, and other methods.

**Results:**

MAGL-18c markedly attenuated hepatic inflammation by suppressing TGF-β/Smad signaling and reducing pro-inflammatory cytokine production. Moreover, MAGL-18c significantly improved liver histopathology, reduced neutrophil infiltration, modulated unsaturated fatty acid metabolism, and alleviated hepatocyte apoptosis and mitochondrial dysfunction.

**Discussion:**

These findings indicate that MAGL-18c protects against LPS-induced SALI through coordinated regulation of inflammation, apoptosis, mitochondrial function, and lipid metabolism, highlighting its potential as a promising therapeutic candidate for sepsis-associated liver injury.

## 1 Introduction

Sepsis is a critical systemic hyper inflammatory condition that leads to extensive tissue damage and multiple organ dysfunction. The World Health Organization reports that the overall survival rate for individuals suffering from sepsis is approximately only 30% (1). Among the various organs that may be affected, liver dysfunction is closely associated with high mortality and poor prognosis, making it a reliable clinical indicator (2, 3). The incidence of Sepsis-associated liver injury (SALI) has been reported to range from 34% to 46%, and mortality in sepsis patients with liver dysfunction or failure can reach 54%–68% (4). Currently, there is no standardized treatment for SALI; most approaches rely on general management of sepsis combined with symptomatic therapy for liver dysfunction (5, 6). Although research on the mechanisms and contributing factors of SALI is increasing, effective drugs targeting specific molecular pathways remain lacking. Therefore, there is an urgent need to develop new therapeutic strategies to alleviate sepsis-induced liver injury.

LPS is a part of the outer membrane of Gram-negative bacteria and can cause both sudden and long-term liver damage (7). It harms the body mainly through inflammation, oxidative stress, cell death, and autophagy (8, 9). MAGL is a serine hydrolase that breaks down the endocannabinoid 2-arachidonoylglycerol (2-AG) and converts monoglycerides into glycerol and fatty acids, which affects inflammation and endocannabinoid balance (10). LPS can increase MAGL activity, speeding up 2-AG breakdown and raising levels of arachidonic acid (AA), which is the source of important molecules like prostaglandins, leukotrienes, and thromboxanes. MAGL is found in many tissues, and about 10–20% of it is in the liver. Drugs that block MAGL have shown benefits in different disease models, including brain disorders and sepsis (11–13). MAGL-18c is a strong MAGL inhibitor, and it has been reported to protect against kidney injury (14). In our tests, it binds to MAGL with a docking energy of −8.9 kcal/mol, and the enzyme assay shows an IC_50_ of 662.6 nM and a maximum inhibition of about 76%, showing it works well and reversibly. With this in mind, we decided to study how MAGL-18c affects liver injury caused by sepsis in mice.

This study aims to investigate the mechanism by which MAGL-18c alleviates LPS-induced SALI. To this end, we performed proteomics and metabolomics analyses on liver tissue to characterize changes in protein expression and small-molecule metabolites. These data were used to analyze the molecular pathways involved in the liver’s response to MAGL-18c during SALI. Collectively, this study reveals the mechanistic basis for MAGL-18c’s hepatoprotective effects, providing a theoretical framework for its potential application in SALI prevention and treatment, as well as future drug development.

## 2 Materials and methods

### 2.1 Survival analysis and sepsis scoring

After intraperitoneal administration of LPS (25 mg/kg), mice were monitored for survival for up to 48 h. Survival was recorded at predetermined intervals, and Kaplan–Meier survival curves were generated. Differences in survival among groups were analyzed using the log-rank (Mantel–Cox) test. Sepsis severity was evaluated at 24 h post-LPS challenge using a standardized murine sepsis scoring system. Clinical parameters, including general appearance, spontaneous activity, level of consciousness, responsiveness to external stimuli, posture, and respiratory pattern, were systematically assessed and scored according to predefined criteria. The individual parameter scores were summed to generate an overall sepsis score, with higher scores indicating greater disease severity. All evaluations were conducted by investigators blinded to group allocation.

### 2.2 Drugs and reagents

MAGL-18c (purity >98%) was obtained from Shandong New Time Pharmaceutical Co., Ltd. (Shandong, China). LPS (L2880) was purchased from Sigma-Aldrich (Shanghai, China). Antibodies against Bax, Bcl-2, and TGF-β1 were from Abcam (Cambridge, UK), while antibodies against Smad2, Smad3, phospho-Smad2, and phospho-Smad3 were obtained from Cell Signaling Technology (Boston, MA, USA). Anti-β-actin antibody, HRP-conjugated secondary antibodies, chemiluminescent HRP substrate, and BeyoECL Plus reagent were purchased from Beyotime Biotechnology (Shanghai, China). PVDF membranes were obtained from Merck Millipore (MA, USA), and Hematoxylin–Eosin (H&E) staining kits were purchased from Solarbio (Beijing, China).

### 2.3 Animals and ethics statement

A total of 50 male C57BL/6J mice (6–8 weeks old, 18–22 g) were obtained from Jinan Pengyue Laboratory Animal Breeding Co., Ltd. (Jinan, China; License No. SYXK (Lu) 2023 0023). Mice were housed under controlled temperature and humidity with a 12-hour light–dark cycle and had free access to water and standard pellet feed (Certificate No. AN-IACUC-2024-096). Animals were fasted overnight prior to experiments, with water available ad libitum.

### 2.4 Establishment of the model of LPS-induced SALI

After a one-week acclimatization, mice were randomly assigned to five groups: control, LPS, and MAGL-18c treatment groups (1, 2.5, and 5 mg/kg). Control and LPS groups received intraperitoneal saline based on body weight, while treatment groups received intraperitoneal MAGL-18c. Sixty minutes later, the control group received saline, whereas the LPS and MAGL-18c groups were injected with LPS (25 mg/kg, i.p.). Mice were sacrificed 24 hours after LPS administration. Anesthesia was induced with haloperidol hydrochloride (7.5 mg/kg, i.m.) and ketamine hydrochloride (100 mg/kg, i.p.), followed by euthanasia via exsanguination from the abdominal vena cava. Blood samples were collected from the main abdominal vein, and liver tissues were harvested and stored at −80°C.

### 2.5 Liver index

The liver was removed and weighed, and the liver index was subsequently calculated using the following formula: Liver Index = Liver Mass (mg)/Mouse Body Weight (g) × 10.

### 2.6 Real-time quantitative polymerase chain reaction

Total RNA was extracted from liver tissue using the RNAeasy™ Animal RNA Extraction Kit (R0027, Beyotime, Shanghai, China), and cDNA was synthesized following the manufacturer’s instructions (D7190M, Beyotime). Quantitative real-time PCR was performed on a 7500 Fast Real-Time PCR System (Thermo, Germany). Relative gene expression was calculated using the 2^−ΔΔCT method. The sequences of primers used for each target gene are listed in **Table 1**.

**Table 1:** Primers used for quantitative real-time PCR analysis.

Gene name	Forward primer (5′ → 3′)	Reverse primer (5′ → 3′)
GAPDH	AGGTCGGTGTGAACGGATTTG	TGTAGACCATGTAGTTGAGGTCA
Bax	TGAAGACAGGGGCCTTTTTG	AATTCGCCGGAGACACTCG
Bcl-2	GTCGCTACCGTCGTGACTTC	CAGACATGCACCTACCCAGC
Caspase 3	TGGTGATGAAGGGGTCATTTATG	TTCGGCTTTCCAGTCAGACTC
IL-10	GCTCTTACTGACTGGCATGAG	CGCAGCTCTAGGAGCATGTG
TGF-*β*	TCTGCATTGCACTTATGCTGA	AAAGGGCGATCTAGTGATGGA

### 2.7 Western blot assay

Liver lysates were separated by 10% SDS–PAGE and transferred onto PVDF membranes. Membranes were blocked with 5% skim milk for 1 h at room temperature, then incubated overnight at 4°C with primary antibodies. After washing, membranes were incubated with HRP-conjugated secondary antibodies at 37°C for 2 h. Protein bands were visualized using enhanced chemiluminescent reagents and imaged with a ChemiScope 6200 system (Clinx Scientific Instruments, Shanghai, China).

### 2.8 Enzyme-linked immunosorbent assay

Levels of ATP, MAGL, CB1, CB2, and 2-AG in liver tissue homogenates were measured using commercial ELISA kits according to the manufacturers’ instructions. The following kits were used: ATP (CLOUD–CLONE, Wuhan, China), MAGL (Shanghai Hengyuan Biotech, China), CB1 (Tianjin Yueteng Biotech, China), CB2 (Shanghai Sentai Biotech, China), and 2-AG (CLOUD–CLONE, Wuhan, China).

### 2.9 Blood biochemistry

The serum was collected and assayed for Alanine Aminotransferase (ALT), Aspartate Aminotransferase (AST), Lactate Dehydrogenase (LDH), Total Cholesterol (TC), and Triglyceride (TG) following the manufacturer’s instructions of the kit (Mindray, Shanghai, China).

### 2.10 Hematoxylin and eosin staining

Thin sections of liver tissues embedded in paraffin were dehydrated using a series of graded ethanol solutions before being stained with H&E. Subsequently, three pathologists assessed the extent of tissue inflammation and damage.

### 2.11 Immunohistochemistry

Liver sections were deparaffinized in xylene and ethanol, followed by antigen retrieval in sodium citrate buffer using high-pressure heating. Endogenous peroxidase activity was blocked with 3% hydrogen peroxide for 10 min. Sections were incubated with 5% normal goat serum for 10 min to reduce nonspecific binding. Primary antibodies (1:1000) were applied overnight at 4°C, followed by incubation with biotin-conjugated secondary antibodies and streptavidin-HRP at room temperature. Immunoreactivity was visualized using diaminobenzidine (DAB) substrate (Beijing Daylight Technology, China), and sections were dehydrated and mounted with neutral resin. Staining intensity and the percentage of positive cells were quantified using ImageJ software (version 1.5.7, NIH).

### 2.12 Cytometric bead array assay

The expression levels of key inflammatory factors, including IL-6, IL-10, IL-1β, and TNF, were assessed using the CBA Mouse Inflammation Kit (BD Biosciences, #558266), following the manufacturer’s instructions.

### 2.13 Examining the influence of MAGL-18c on liver tissue homogenate metabolism caused by LPS via unbiased LC-MS analysis

Approximately 50 mg of liver tissue was weighed and placed into Eppendorf tubes containing internal standards. Ice-cold methanol:acetonitrile:water (2:2:1, v/v) was added, and two steel balls were included for homogenization. Samples were vortexed, oscillated, and sonicated, followed by incubation at −50°C for 60 min and centrifugation at 12,000 rpm for 15 min at 4°C. A 500 µL aliquot of the supernatant was transferred to a new tube, centrifuged again, and equilibrated at 20°C for 2 h. Metabolic profiling was performed using a Dionex Ultimate 3000 RS UPLC system coupled to a Q Exactive quadrupole-orbitrap mass spectrometer with a heated electrospray ionization source (Thermo Fisher Scientific, Waltham, MA, USA).

### 2.14 Transmission electron microscopy

Liver tissues were immediately fixed in 2.5% glutaraldehyde at 4°C for at least 4 h, washed with phosphate-buffered saline (PBS, pH 7.4), and post-fixed in 1% osmium tetroxide for 1 h. Samples were dehydrated through a graded ethanol series (30%–100%), infiltrated with acetone, and embedded in Epon 812 resin (SPI, 02660-AB). Ultrathin sections (~70 nm) were cut using an ultramicrotome (Leica UC7), double-stained with uranyl acetate and lead citrate, and examined under a transmission electron microscope (HT7800, HITACHI, Japan) to assess mitochondrial ultrastructure.

### 2.15 Proteomics research

Approximately 50 mg of frozen liver tissue was placed in a grinding tube and lysed with 8 M urea, 1% SDS, and protease inhibitors. The samples were homogenized using a cryogenic grinder (3 × 3 min) and sonicated at low temperature for 30 minutes. The lysates were then centrifuged at 14,000 g and 8°C for 15 minutes, and the supernatant was collected for protein quality assessment. After confirming the sample quality, the proteins were digested into peptides and analyzed using data-independent acquisition mass spectrometry (DIA-MS). Proteomic data were analyzed using the Majorbio Cloud platform (cloud.majorbio.com). Differential protein expression was determined using the t-test in R, with P < 0.05 considered significant and fold change (FC) > 2 defining markedly altered proteins. Metabolic pathway analysis was performed using the KEGG database (genome.jp/kegg/).

### 2.16 Medium/long-chain fatty acid profiling

Approximately 50 mg of liver tissue was placed in a 2 mL grinding tube with a steel bead and 1 mL dichloromethane:methanol (1:1, v/v). Samples were homogenized in a cryogenic grinder at 50 Hz for 3 min, sonicated at low temperature for 15 min, and incubated at −20°C for 15 min. After centrifugation at 13,000 × g and 4°C for 10 min, 500 µL of the supernatant was transferred to a 1.5 mL tube and evaporated under nitrogen. The dried extract was derivatized with 0.5 mL of 0.5 M sodium hydroxide in methanol, vortexed for 30 s, sonicated at 4°C for 10 min, and incubated at 60°C for 30 min. After cooling, 0.5 mL n-hexane was added, vortexed, centrifuged (13,000 × g, 4°C, 10 min), and 100 µL of the upper n-hexane layer was transferred to a vial for GC–MSD analysis (Agilent Technologies, CA, USA).

### 2.17 Statistical analysis

All data were expressed as the average ± SD. Differences among the various groups were assessed using one-way analysis of variance (ANOVA), followed by a *post hoc* Bonferroni correction for multiple comparisons. Statistical significance is defined as *P* < 0.05.

## 3 Results

### 3.1 Systemic protective effects of MAGL-18c: survival benefit and clinical symptom improvement

**Figure 1:**
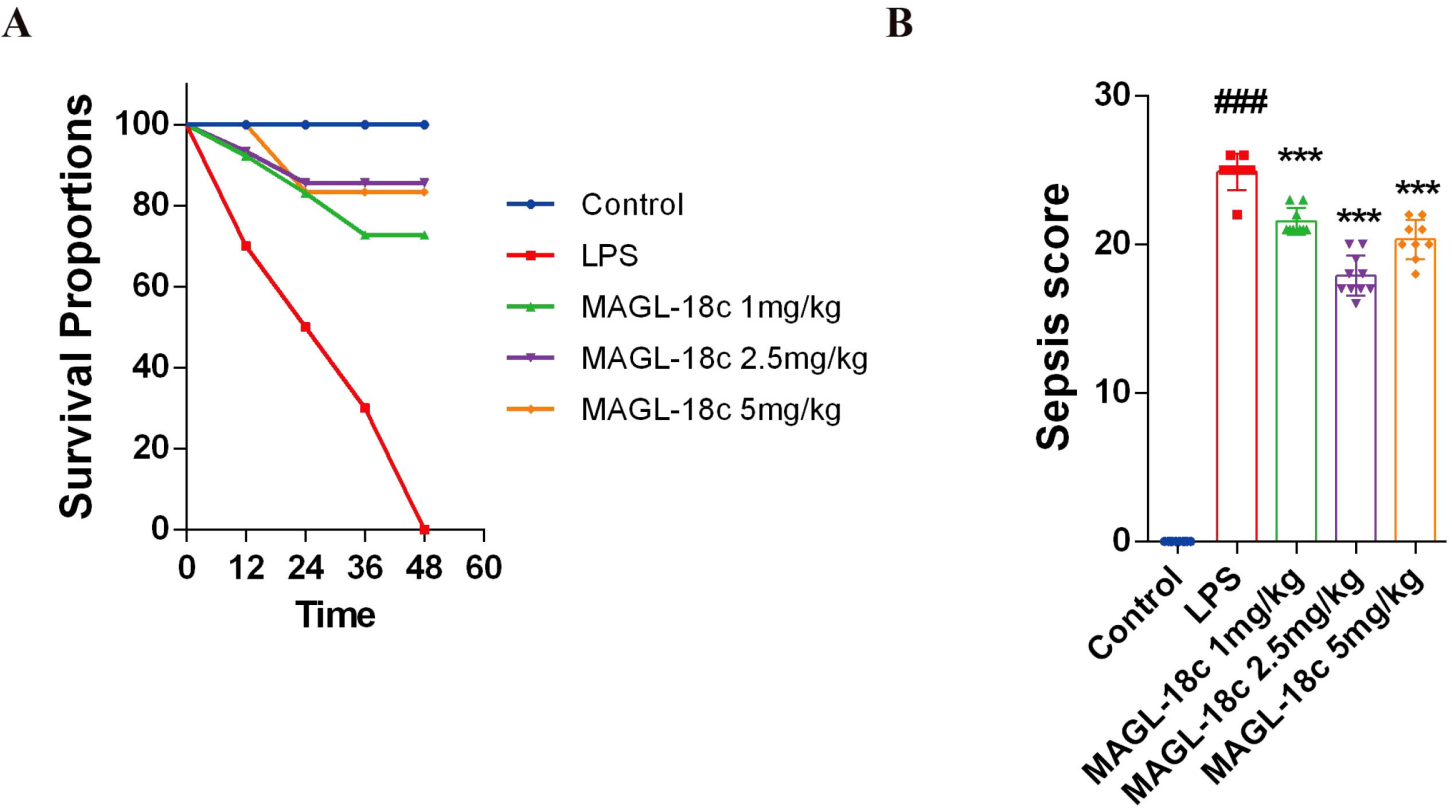
Effects of MAGL-18c on survival rate and disease severity in LPS-induced sepsis mice. **(A)** Survival Analysis. Mice were pretreated with MAGL-18c or saline before receiving a 2.5 mg/kg LPS challenge. **(B)** Sepsis Clinical Scoring. Blinded clinical assessments were conducted 24 hours after LPS challenge to evaluate sepsis severity. Scoring criteria included coat condition, activity level, respiration, ocular appearance, and responsiveness to stimuli. Survival data were analyzed using the Log-rank test. Clinical scores are presented as mean ± standard deviation. All data are expressed as mean ± SD; control group, #P < 0.05, ##P < 0.01, ###P < 0.001; LPS group, *P < 0.05, **P < 0.01, ***P < 0.001.

To validate whether MAGL-18c can prevent LPS-induced sepsis, we assessed mouse survival rates and disease severity. Following high-dose LPS (2.5 mg/kg) treatment, all saline-treated mice died within 72 hours. In contrast, pretreated MAGL-18c mice exhibited significantly improved survival, with approximately 80% surviving beyond 72 hours (Log-rank test, p < 0.01; **Figure 1A**). Clinical severity scores assessed 24 hours post-LPS injection revealed marked deterioration in LPS-treated mice across hair condition, locomotor activity, respiratory status, ocular appearance, and responsiveness. MAGL-18c pretreatment significantly reduced these scores (two-way ANOVA, p<0.05), manifesting as improvements in fur condition, mobility, and respiratory function (**Figure 1B**).

These findings indicate that MAGL-18c not only delays sepsis progression but also ameliorates overall clinical symptoms and enhances survival rates, suggesting its potential as an effective therapeutic agent for sepsis-related complications.

### 3.2 MAGL-18c alleviates LPS-induced SALI in mice

**Figure 2:**
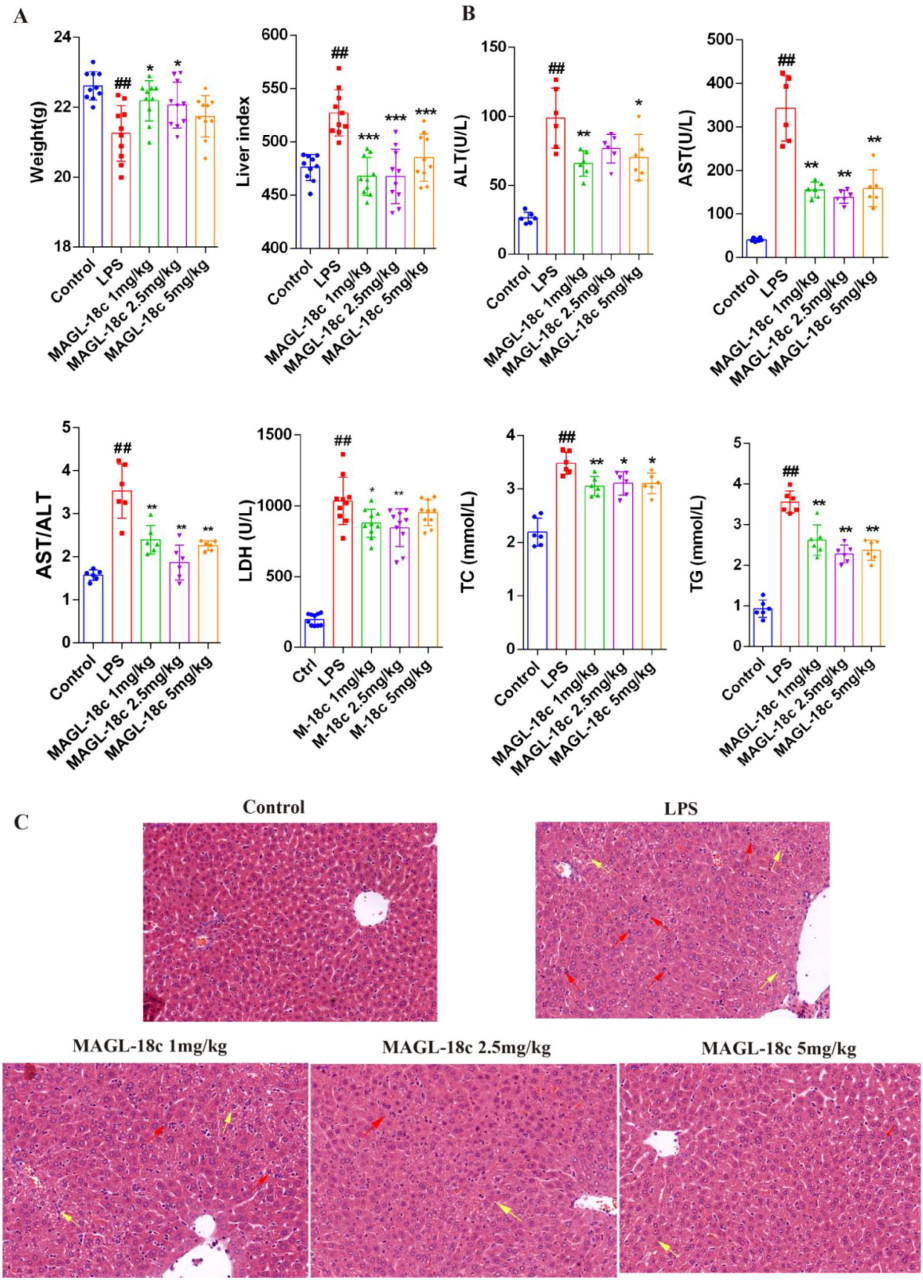
Effects of MAGL-18c on LPS-induced 24-hour SALI in mice. **(A)** Changes in body weight and liver index in LPS-treated mice at 24 hours (n=10). **(B)** Serum levels of ALT, AST, AST/ALT ratio, LDH, TC, and TG (n=6). Data were analyzed using ANOVA with Tukey’s *post hoc* test. **(C)** Representative histopathological images of liver tissue (20× magnification, 50 μm scale bar). Red arrows indicate inflammatory cell infiltration, while yellow arrows highlight lipid vacuoles of varying sizes. Data analysis was performed with Tukey’s *post hoc* test following one-way ANOVA. All data are expressed as mean ± SD; control group, ##P < 0.01; LPS group, *P < 0.05, **P < 0.01, ***P < 0.001.

To evaluate the hepatoprotective effects of MAGL-18c, we examined liver-related parameters following LPS challenge. LPS caused significant body weight loss and increased liver indices compared to controls, indicating systemic inflammation and hepatomegaly. MAGL-18c, particularly at 2.5 mg/kg, mitigated weight loss and lowered liver indices (**Figure 2A**). Serum ALT, AST, and LDH levels were markedly elevated in LPS-treated mice (~3–4 folds versus control), reflecting substantial liver injury, whereas MAGL-18c treatment significantly reduced these enzymes, with the 2.5 mg/kg dose showing the strongest effect (**Figure 2B**). MAGL-18c also attenuated LPS-induced increases in serum total cholesterol (TC) and triglycerides (TG).

Histological evaluation using H&E staining showed normal hepatic architecture in controls, while LPS induced extensive inflammatory infiltration and steatosis. MAGL-18c treatment improved these pathological features, especially at 2.5 mg/kg, restoring hepatocyte arrangement, reducing inflammation, and improving nuclear morphology (**Figure 2C**). These results demonstrate that MAGL-18c provides effective protection against LPS-induced liver injury.

### 3.3 MAGL-18c attenuates LPS-induced inflammatory cytokine production and neutrophil infiltration

**Figure 3:**
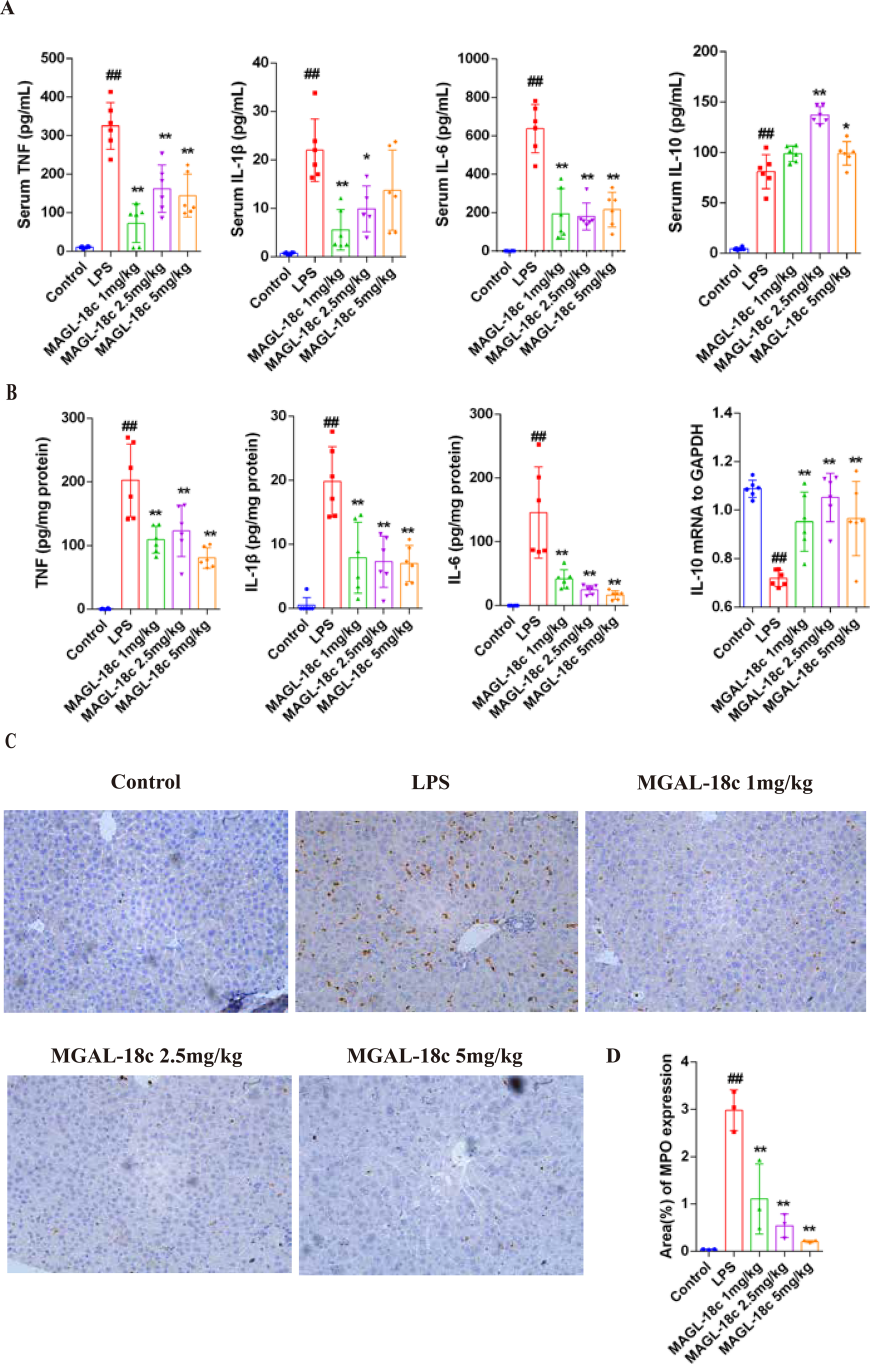
The anti-inflammatory effect of MAGL-18c on LPS-induced acute liver injury (ALI) in mice. **(A)** Levels of TNF, IL-1*β*, IL-6 and IL-10 in serum (n=6). **(B)** Levels of TNF, IL-1*β* and IL-6 in liver tissue homogenates detected by CBA and the relative mRNA expression levels of IL-10. **(C)** The results of quantitative analysis of the MPO positive area (n=6). Image analysis was completed using ImageJ software. Analysis utilized Tukey’s *post hoc* test following one-way ANOVA. All data are expressed as the mean ± SD; control group, ^#^*P* < 0.05, ^##^*P* < 0.001; LPS group, ^*^P < 0.05, ^**^P < 0.01. **(D)** The expression of MPO in mouse liver tissue was detected by immunohistochemistry (n=6). Representative images were shown for the control group, the LPS group, and the MAGL-18c group. DAB staining was brown-yellow, and the cell nuclei were counterstained with hematoxylin. Scale bar = 50*μ*m.

To evaluate the anti-inflammatory effect of MAGL-18c, serum and liver levels of inflammatory cytokines were measured. LPS markedly increased pro-inflammatory cytokines TNF, IL-1β, and IL-6, while IL-10 levels were elevated in serum but reduced at the mRNA level, likely reflecting a compensatory anti-inflammatory response (**Figures 3A, B**). MAGL-18c treatment (with the 2.5 mg/kg dose showing the most effective results overall) significantly reduced serum and liver homogenate levels of TNF, IL-1β, and IL-6, while increasing IL-10 levels.

Immunohistochemical staining for myeloperoxidase (MPO) showed marked neutrophil infiltration in livers from LPS-induced mice, as evidenced by increased MPO positive areas. Treatment with MAGL-18c reduced MPO expression in a dose-dependent manner, with levels approaching those of controls at 2.5 and 5 mg/kg (**Figures 3C, D**), indicating effective suppression of neutrophil-driven hepatic inflammation.

### 3.4 MAGL-18c inhibits the apoptosis caused by LPS-induced mitochondrial dysfunction

**Figure 4:**
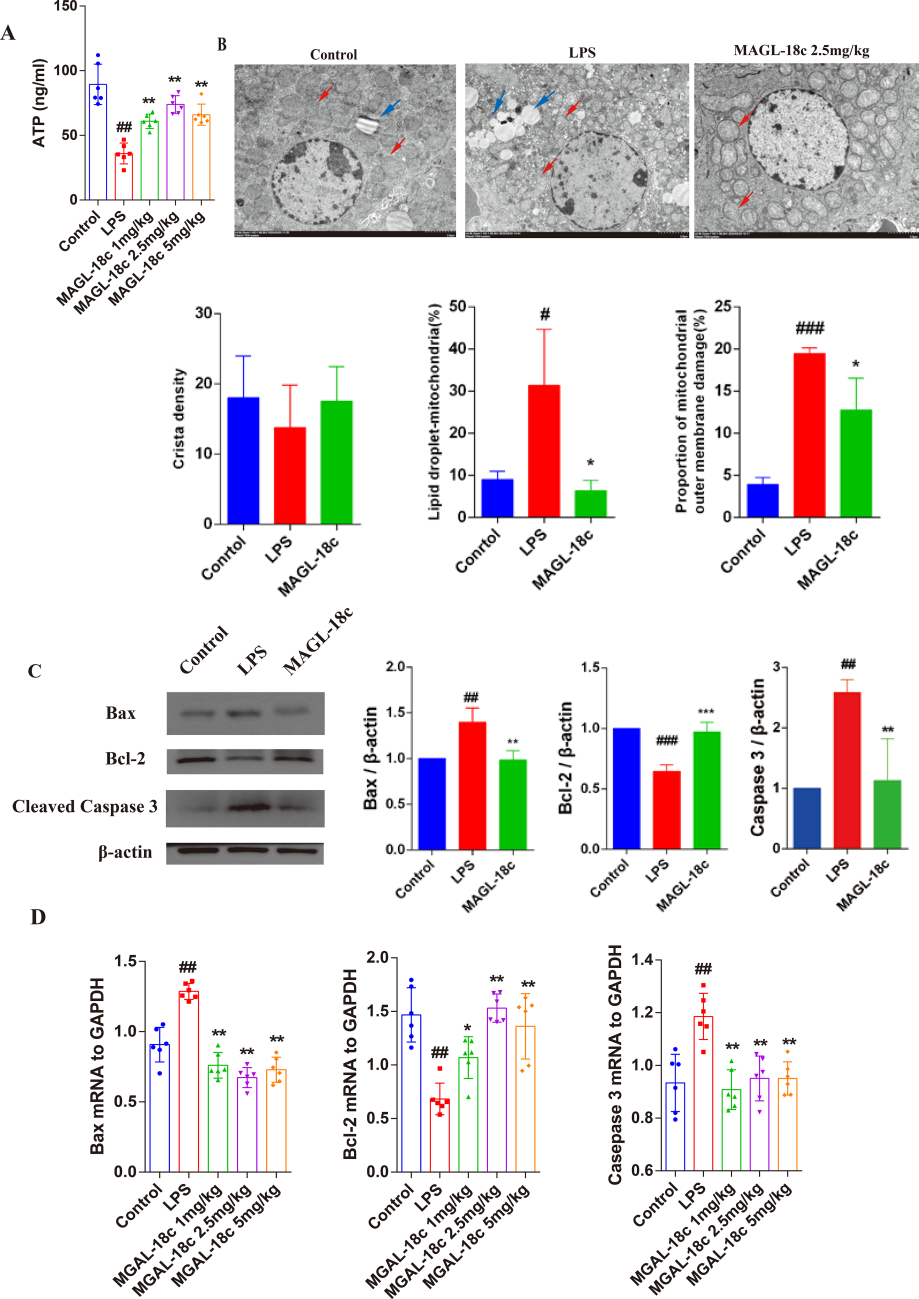
Effects of MAGL-18c on LPS-induced mitochondrial dysfunction and apoptosis in the liver. **(A)** ATP content measured in liver tissue homogenates. **(B)** Representative transmission electron microscopy (TEM) images and quantitative results for ridge density, proportion of mitochondria with damaged outer membranes, and lipid droplet-associated mitochondria. **(C)** Western blot analysis of apoptosis-related proteins, including Bax, Bcl-2, and Cleaved-Caspase-3, with corresponding quantitative densitometry. **(D)** The mRNA expression levels of Bax, Bcl-2, and Caspase-3 assessed by qPCR. Data were analyzed using one-way ANOVA followed by Tukey’s *post hoc* test. All data are expressed as mean ± SD; control group, #P < 0.05, ##P < 0.01, ###P < 0.001; LPS group, *P < 0.05, **P < 0.01, ***P < 0.001.

To evaluate the impact of MAGL-18c on mitochondrial function and apoptosis in LPS-induced SALI, hepatic ATP levels were quantified (**Figure 4A**). LPS treatment markedly decreased ATP content compared with controls, whereas MAGL-18c restored ATP, with the 2.5 mg/kg dose showing the strongest effect. Based on the pharmacodynamic results, this dose was selected for all subsequent experiments.

Transmission electron microscopy revealed mitochondrial cristae disruption and fragmentation in the LPS group, with some mitochondria associated with lipid droplets (**Figure 4B**). Quantitative analysis showed decreased cristae density, increased lipid droplet-associated mitochondria, and higher outer membrane rupture rates in LPS-treated livers, all of which were significantly improved by MAGL-18c.

Consistent with mitochondrial protection, LPS upregulated pro-apoptotic Bax and cleaved Caspase-3 and downregulated anti-apoptotic Bcl-2, whereas MAGL-18c reversed these changes at both protein and mRNA levels (**Figures 4C, D**), indicating that MAGL inhibition mitigates mitochondrial dysfunction-induced hepatocyte apoptosis.

### 3.5 MAGL-18c alleviates LPS-induced SALI through TGF-*β*/Smad pathway in proteomics

**Figure 5:**
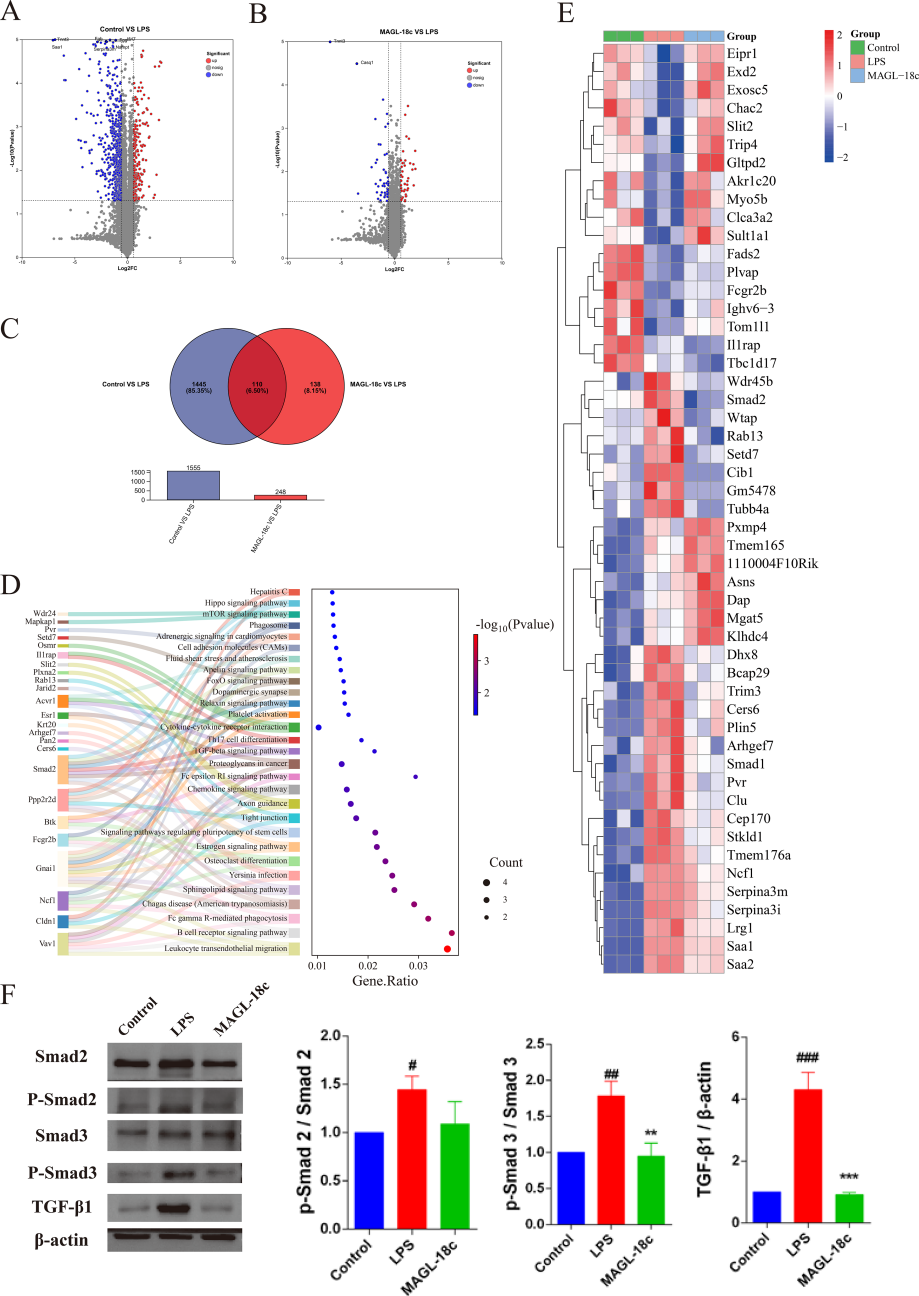
MAGL-18c inhibits TGF-*β/*Smad signaling pathway in liver tissue. **(A)** Volcano plots compared control group and LPS group. **(B)** Volcano plots compared MAGL-18c and LPS. **(C)** The total of 1445 proteins changed in control group compared with LPS group, 138 proteins changed in MAGL-18c group compared with LPS group, and 110 proteins coincided. **(D)** KEGG pathway analysis. **(E)** Heatmap of differential proteins among three groups. **(F)** Representative Western blot images of Smad2, Smad3, P-Smad2, P-Smad3, TGF-β1and β-actin are shown (left panels). Quantifications of band intensities normalized are shown (right panels).All data are expressed as mean ± SD; control group, #P < 0.05, ##P < 0.01, ###P < 0.001; LPS group, **P < 0.01, ****P < 0.0001.

To investigate the molecular mechanisms of MAGL-18c in sepsis-induced liver injury, label-free quantitative proteomics was performed on liver tissues. Volcano plots (**Figures 5A, B**) showed 1,455 differentially expressed proteins (DEPs) between control and LPS groups, and 138 DEPs between MAGL-18c and LPS groups, indicating partial reversal of LPS-induced proteomic changes. A Venn diagram identified 110 overlapping DEPs significantly altered by LPS and modulated by MAGL-18c (**Figure 5C**).

KEGG pathway analysis of these overlapping DEPs revealed significant enrichment in inflammation- and metabolism-related pathways, notably the TGF-β/Smad signaling pathway, highlighting its role in SALI pathogenesis and resolution (**Figure 5D**). Hierarchical clustering showed distinct protein expression patterns, with MAGL-18c partially restoring normal levels (**Figure 5E**).

Proteomics indicated upregulation of TGF-β1 and phosphorylated Smad2/3 in LPS-treated livers, which was confirmed by Western blot. MAGL-18c treatment significantly reduced TGF-β1 and p-Smad2/3 expression (**Figure 5F**), suggesting that modulation of the TGF-β/Smad pathway contributes to its hepatoprotective effects.

### 3.6 MAGL-18c regulates liver metabolic disorders caused by LPS

**Figure 6:**
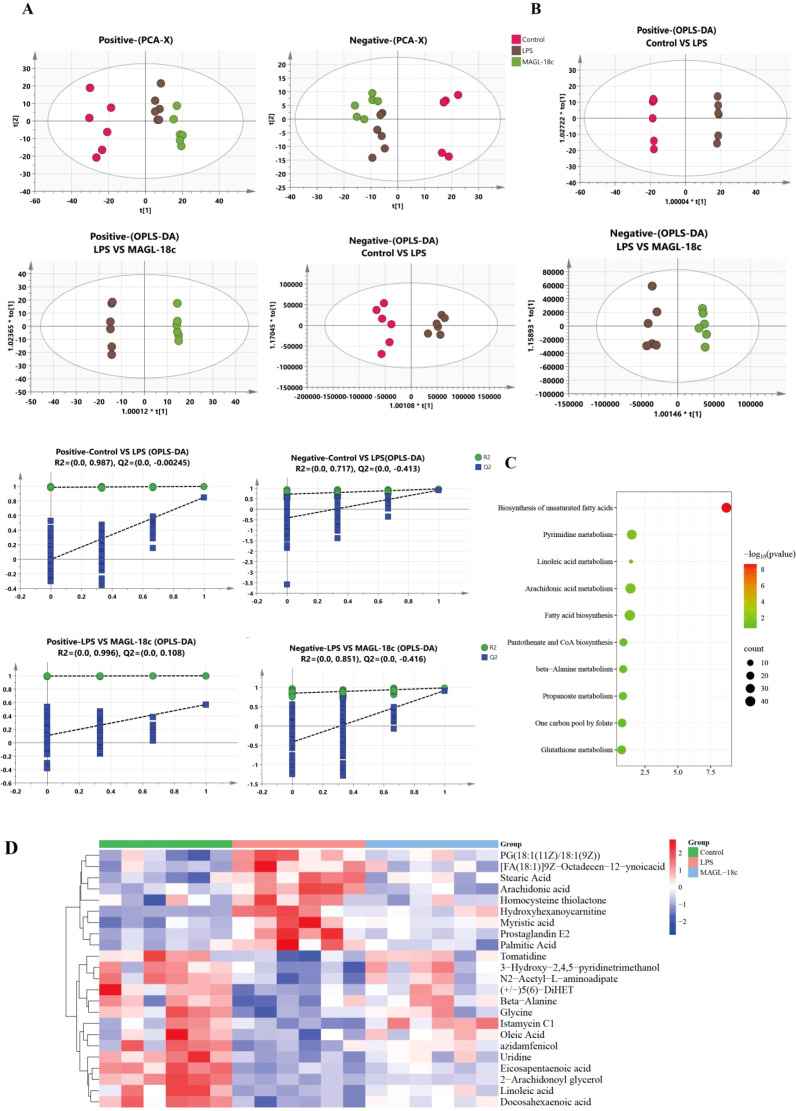
Effects of MAGL-18c on liver metabolites in ALI mice. **(A)** PCA analysis (n=6). **(B)** OPLS-DA analysis (n=6). **(C)** Heat map analysis of differential metabolites (n=6). **(D)** KEGG analysis of serum metabolic pathways.

To explore the metabolic changes associated with LPS-induced liver injury and the effect of MAGL-18c, untargeted metabolomic profiling of liver tissue was performed. PCA and OPLS-DA analysis revealed distinct metabolic profiles among the control, LPS, and MAGL-18c groups (**Figures 6A, B**). Then, VIP>1 and *P* < 0.05 were used as screening conditions for differential metabolites.

A total of 24 differential metabolites were identified between the LPS and control groups, primarily consisting of medium- and long-chain fatty acids (MCFAs and LCFAs). These metabolites were analyzed using MetaboAnalyst 6.0, revealing five significantly altered pathways: biosynthesis of unsaturated fatty acids, pyrimidine metabolism, linoleic acid metabolism, arachidonic acid metabolism, and fatty acid biosynthesis (**Figures 6C, D**). These findings indicate that MAGL-18c mitigates LPS-induced metabolic disturbances, particularly in lipid-related pathways.

### 3.7 MAGL-18c restores hepatic MCFAs and LCFAs homeostasis in LPS-induced SALI

**Figure 7:**
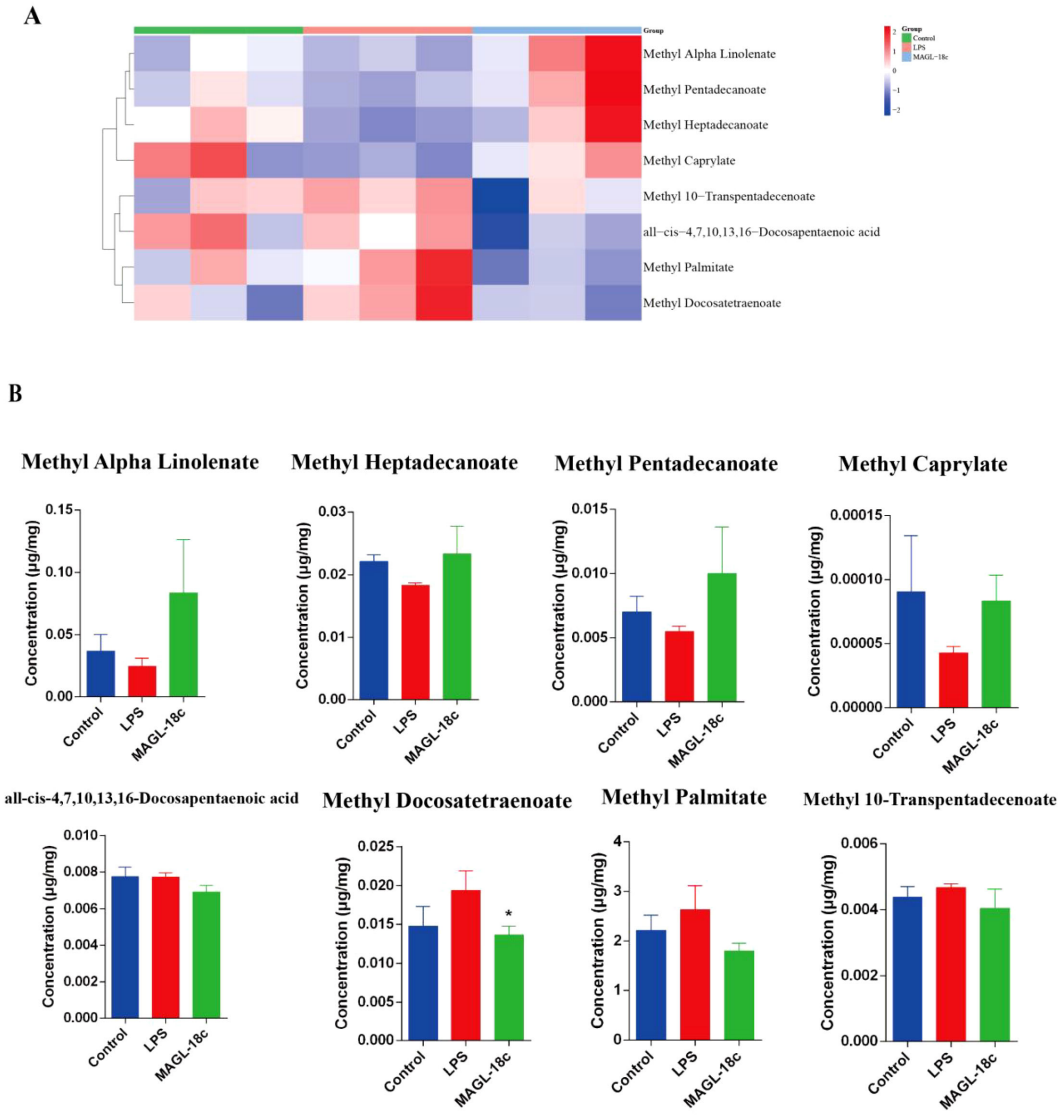
Analysis of MCFAs and LCFAs composition in liver tissue homogenates from mice in each treatment group 24 hours after LPS modeling (detected by GC-MS). **(A)** Heatmap of trending MCFAs and LCFAs among three groups. **(B)** Content of partial MCFAs and LCFAs in liver tissues. All data are expressed as the mean ± SD; LPS group, ^*^P < 0.05.

To examine alterations in MCFAs and LCFAs during SALI, GC-MS analysis was conducted. Heatmap clustering analysis revealed a trend toward fatty acid profile differences among groups (**Figure 7A**), reflecting broad lipid metabolic changes. MAGL-18c partially restored several long-chain polyunsaturated fatty acids, indicating a trend toward metabolic normalization. LPS increased pro-inflammatory fatty acids, such as palmitic acid, while reducing anti-inflammatory species like pentadecanoic acid. Although changes in MCFA and LCFA levels did not reach statistical significance, MAGL-18c showed a consistent trend toward improved lipid metabolism (**Figure 7B**). Together with reduced hepatic lipid droplets and enhanced fatty acid oxidation markers, these findings suggest that MAGL-18c may help restore liver lipid balance.

### 3.8 MAGL-18c-mediated bidirectional regulation of 2-AG/CB and AA/PGE2

**Figure 8:**
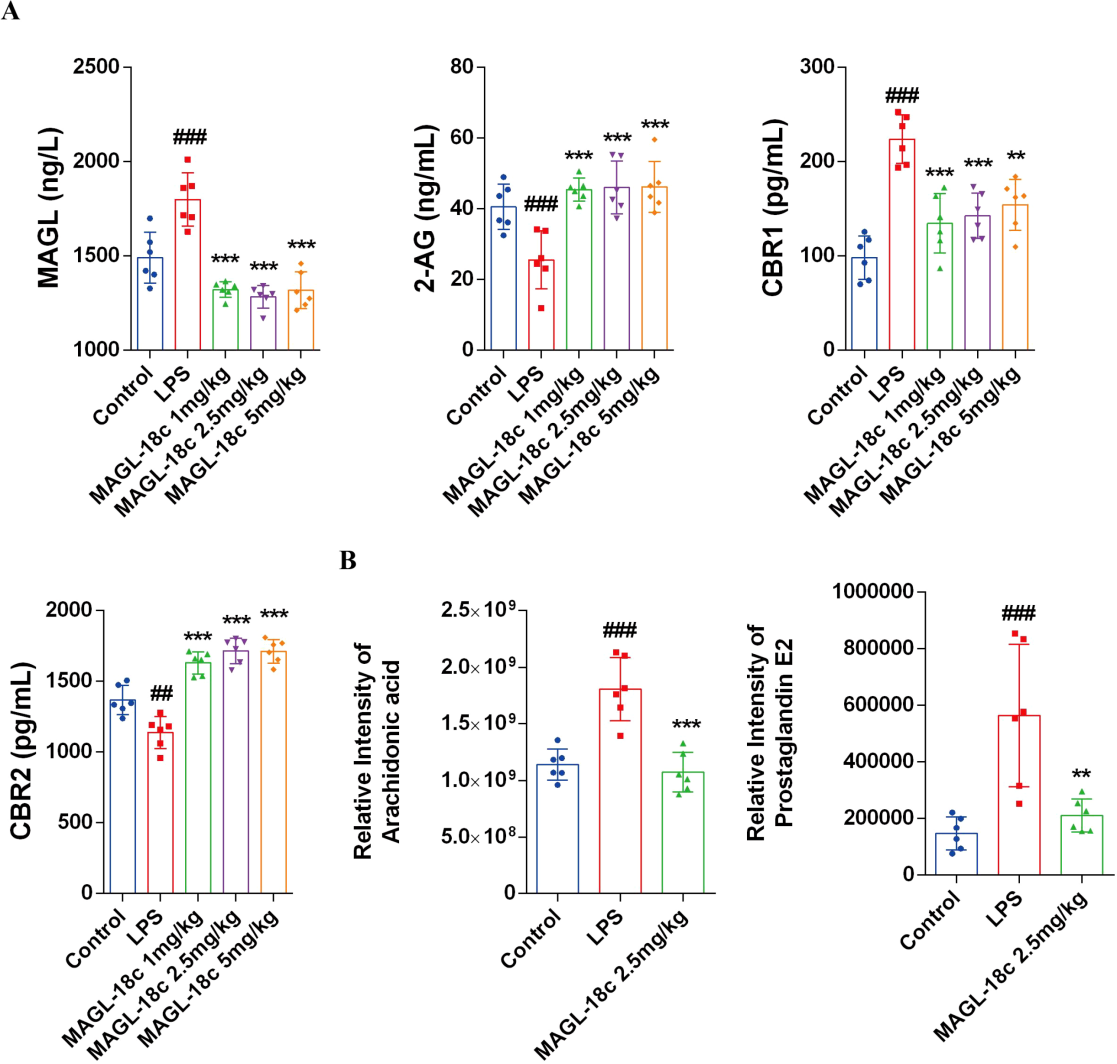
MAGL-18c modulates the “2-AG-AA axis” to promote anti-inflammatory effects and suppress pro-inflammatory actions. **(A)** Levels of MAGL, 2-AG, CBR1, and CBR2 in liver tissue homogenates. **(B)** Relative Intensity of AA and PGE2 in liver tissue homogenates. All data are expressed as mean ± SD; control group, ##P < 0.01,###P < 0.001; LPS group, **P < 0.01, ***P < 0.001.

To explore the mechanism of MAGL-18c, we first examined its effects on the MAGL–2-AG–cannabinoid receptor axis (**Figure 8**). All MAGL-18c dose groups suppressed LPS-induced MAGL activity, resulting in accumulation of its substrate 2-AG. The increase in 2-AG selectively upregulated CB2 receptor expression while downregulating CB1 receptor expression, highlighting a targeted modulation of the endocannabinoid pathway.

Subsequently, we conducted an in-depth analysis of the metabolomics results and discovered significant alterations in downstream pro-inflammatory mediators. MAGL-18c treatment reduced arachidonic acid (AA) production and markedly suppressed PGE_2_ levels. These results indicate that MAGL-18c limits AA/PGE_2_-driven inflammatory signaling, suggesting that its protective effect in the liver involves both enhancement of 2-AG/CB2 signaling and inhibition of AA-mediated pro-inflammatory responses.

## 4 Discussion

SALI is a key pathogenic factor contributing to multiple organ dysfunction and high mortality in sepsis patients. Although inflammation is a core pathophysiological mechanism (15), it alone cannot fully explain the progression of organ failure. Emerging evidence indicates that metabolic dysregulation, systemic inflammation, and apoptosis are interlinked, collectively driving liver injury. Notably, inflammation-driven hepatocyte apoptosis and disrupted metabolic homeostasis play pivotal roles in disease progression (16). Our study demonstrates that MAGL-18c exerts significant protective effects against mouse SALI by targeting multiple critical nodes within the pathological network: preserving mitochondrial structure and function, regulating the TGF-β/Smad pathway, and reshaping fatty acid metabolism.

MAGL-18c treatment substantially enhanced survival, improved liver function, and ameliorated histopathological damage. In LPS-challenged mice, survival reached approximately 80% at 72 hours, compared with complete mortality in untreated controls, while clinical sepsis scores reflected a reduction in disease severity. Key indicators of liver injury, including serum AST, ALT, and LDH (17, 18), were significantly lowered, accompanied by decreased neutrophil infiltration and MPO activity (19, 20). Concurrently, proinflammatory cytokines (IL-1β, IL-6, TNF) were reduced, whereas IL-10 levels increased in both serum and liver tissue, indicating a shift toward an anti-inflammatory state.

Beyond its anti-inflammatory effects, MAGL-18c markedly preserved mitochondrial structure and function. Sepsis is known to impair mitochondrial integrity and cellular bioenergetics (21–23); however, MAGL-18c treatment maintained mitochondrial morphology, elevated hepatocyte ATP levels, and reduced membrane damage and lipid droplet-related abnormalities. Serum TC and TG levels were also lowered, reflecting improved mitochondrial performance and lipid handling. These findings indicate that MAGL-18c helps restore hepatocyte bioenergetic capacity, which is critical for meeting the high energy demands during sepsis (24, 25).

Apoptosis and mitochondrial dysfunction are closely linked in sepsis-induced liver injury. LPS-induced lipid metabolism abnormalities increase lipid droplet formation and cause lipotoxicity, impairing mitochondrial integrity and triggering apoptosis (26–28). This process involves Bax activation, Caspase-3 cleavage, and Bcl-2 downregulation, further worsening hepatocyte injury. MAGL-18c restores fatty acid metabolism, reduces lipid toxicity, and supports mitochondrial function, while simultaneously increasing Bcl-2 levels and decreasing Bax and activated Caspase-3, thereby suppressing mitochondrial-mediated apoptosis. This combined effect of MAGL inhibition, reduced inflammation, and restored mitochondrial function provides a mechanistic basis for its protective role in sepsis-associated liver injury.

Proteomic analysis indicated marked enrichment of the TGF-β/Smad signaling pathway, which was abnormally activated in SALI and attenuated by MAGL-18c. LPS-induced inflammatory mediators increased TGF-β1 expression and Smad2/3 phosphorylation (29–32), promoting hepatocyte apoptosis and impairing energy metabolism by suppressing mitochondrial biogenesis and fatty acid oxidation (33–35). This mitochondrial dysfunction can further amplify TGF-β signaling, creating a positive feedback loop that exacerbates inflammation and cell death. MAGL-18c interrupts this loop, providing a protective effect that supports hepatocyte survival and facilitates liver recovery.

Hepatic lipid accumulation is common in sepsis and contributes to liver injury by activating non-oxidative metabolic pathways and generating lipotoxic metabolites that damage mitochondria and trigger apoptosis (36–39). MAGL inhibition has shown anti-inflammatory effects in various disease models (11, 40). In our study, MAGL-18c improved serum biochemical markers, reduced lipid droplet–associated mitochondrial defects, and reshaped hepatic fatty acid profiles, including MCFAs and LCFAs. Although some changes did not reach statistical significance, they offer meaningful hypotheses for future investigation.

Mechanistically, MAGL-18c connects lipid metabolism with inflammation control. By modulating the endocannabinoid system and fatty acid availability, it enhances cellular energy production, selectively upregulates CB2 receptor signaling, and suppresses pro-inflammatory CB1 activity, thereby attenuating TGF-β–related transcriptional responses (41). Combined with ELISA measurements of MAGL activity, its substrate 2-AG, CB1/CB2 receptor expression, and metabolomics data, these findings suggest that MAGL-18c coordinates metabolic and immune pathways, linking lipid homeostasis to inflammation regulation and conferring protection against sepsis-associated liver injury.

In summary, in LPS-induced SALI, TGF-β signaling, mitochondrial apoptosis, and lipid metabolism disturbances form a tightly interconnected network. MAGL-18c disrupts this cycle by acting on multiple nodes, restoring hepatic homeostasis, and providing broad protective effects. This multi-pathway regulatory mechanism underscores the therapeutic potential of MAGL inhibitors in sepsis-associated liver injury.

This study primarily evaluated early-stage (24-hour) liver injury in the LPS model; long-term effects require validation in the CLP model or chronic liver injury settings. Due to experimental limitations, MAGL-18c was not directly compared with other MAGL inhibitors, and its selectivity, kinetic properties, and potential off-target effects warrant further investigation. Nevertheless, its regulation of 2-AG levels and CB2 signaling suggests MAGL inhibition may represent the primary mechanism of action.

## 5 Conclusion

In conclusion, our study reveals that MAGL-18c protects against SALI through a unified mechanism. By inhibiting MAGL, MAGL-18c simultaneously dampens hyperinflammation, suppresses maladaptive TGF-β signaling, protects mitochondrial structure and function, reduces apoptotic signaling, and restores lipid metabolism. These findings highlight its potential as a therapeutic intervention in sepsis, particularly by targeting the TGF-β axis and metabolic reprogramming.

## Data Availability

The datasets presented in this study can be found in online repositories. The names of the repository/repositories and accession number(s) can be found in the article/**Supplementary Material**.
